# Durability of the deltamethrin-treated polypropylene long-lasting net LifeNet® in a pyrethroid resistance area in south western Benin: A phase III trial

**DOI:** 10.1371/journal.pone.0291755

**Published:** 2023-09-20

**Authors:** Armel Djènontin, Daleb Alfa, Aziz Bouraima, Christophe Soares, Amal Dahounto, Sylvie Cornélie, Marc Egrot, Georgia Damien, Franck Remoué, André Barembaye Sagna, Nicolas Moiroux, Cédric Pennetier

**Affiliations:** 1 Centre de Recherche Entomologique de Cotonou (CREC), Cotonou, Bénin; 2 Centre de Recherche Pour la Lutte Contre les Maladies Infectieuses Tropicales (CReMIT), Université D’Abomey-Calavi (UAC), Cotonou, Bénin; 3 MIVEGEC, Univ Montpellier, CNRS, IRD, Montpellier, France; Freelance Consultant, Myanmar, MYANMAR

## Abstract

**Background:**

Long-lasting insecticidal bed nets (LLINs) are a key measure for preventing malaria and their evaluation is coordinated by the World Health Organization Pesticide Evaluation Scheme (WHOPES). LifeNet^®^ was granted WHOPES time-limited interim recommendation in 2011 after successful Phase I and Phase II evaluations. Here, we evaluated the durability and community acceptance of LifeNet^®^ in a Phase III trial from June 2014 to June 2017 in Benin rural area.

**Methods:**

A prospective longitudinal, cluster-randomized, controlled trial with households as the unit of observation was designed to assess the performance of LifeNet® over a three-year period, using a WHOPES fully recommended LLIN (PermaNet^®^ 2.0) as a positive control. The primary outcomes were the bioassay performance using WHO cone assays and tunnel tests, the insecticide content and physical integrity.

**Results:**

At baseline, 100% of LLINs were within the tolerance limits of their target deltamethrin concentrations. By 36 months only 17.3% of LifeNet^®^ and 8.5% of PermaNet^®^ LLINs still were within their target deltamethrin concentrations. Despite these low rates, 100% of both LLINs meet WHO efficacy criteria (≥ 80% mortality or ≥ 95% knockdown or tunnel test criteria of ≥ 80% mortality or ≥ 90% blood-feeding inhibition) after 36 months using WHO cone bio-assays and tunnel tests. The proportion of LLINs in good physical condition was 33% for LifeNet^®^ and 29% for PermaNet^®^ after 36 months. After 36 M the survivorship was 21% and 26% for LifeNet® and PermaNet® respectively. Although both LLINs were well accepted by the population, complaints of side effects were significantly higher among LifeNet^®^ users than PermaNet^®^ ones.

**Conclusion:**

LifeNet^®^ LLINs did meet WHO criteria for bio-efficacy throughout the study period and were well accepted by the population. This is an important step towards getting a full WHO recommendation for use in malaria endemic countries.

## 1. Introduction

Vector control remains by far the most efficient approach for controlling malaria transmission. The estimated number of averted malaria cases between 2000 and 2015 was 663 million of which 68% are attributed to long-lasting insecticidal bed nets (LLINs) [1]. However, global progress on malaria reductions have been slowed since 2015, with only <2% of decline in the malaria incidence between 2015 and 2019, and even an increase in some settings [2]. In Benin, malaria cases have been increasing since 2014. The number of reported malaria confirmed cases has increased from 2 122 011 with 1869 deaths in 2014 to 3 084 525 malaria cases and 2539 deaths in 2019 [2]. This highlights not only the need to strengthen and improve current measures but also to develop complementary tools to further reduce the burden of malaria and contain the spread of resistant malaria vectors.

LLINs remain the cornerstone of malaria prevention [3]. They are made of a material in which an insecticide is incorporated or bound around the fibers so that they retain their biological activity for at least 3 years under field conditions [4]. National malaria control programs (NMCPs) have indeed widely distributed LLINs in sub-Saharan Africa. The percentage of the population with access to an LLIN increased from 33% in 2010 to 56% in 2017 [5]. Even if household surveys indicate that 96% of persons with access to an LLIN use it [6], the actual number would be lower [7]. For example, in a randomized controlled trial in Benin, the actual use of LLINs (i.e. observed on to the sleeping place during the survey) was less than 50% [8]. Among several reasons leading to this low rate of LLIN use, sleeping discomfort under LLINs [9] hot temperatures and low mosquito nuisance are the most mentioned reasons [10].

Pyrethroids are the main WHOPES recommended insecticide for net impregnation due to their high efficacy, their fast acting effect at low dose, and their low toxicity for mammals [11]. With a growing pyrethroid resistance in malaria vectors, it becomes urgent to develop vector control tools capable of controlling resistant malaria vectors [12]. Unfortunately, manufacturers estimate that developing a new active ingredient may take at least 10 years and its cost might reach $300 million. For these reasons, the development of new LLINs based on existing pyrethroids used alone or in combination with a synergist, another class of insecticide with different mode of action and Insect Growth Regulator (IGR) to impregnate polyethylene, polyester or alternative materials is strongly encouraged [13, 14]. Recently, new generation of nets made with polyester and impregnated with two chemical products have shown good efficacy in terms of reduction of infection in children [15]. In addition to this net, there are pyrethroid + PBO treated nets that have also shown good efficacy [16–18].

Durability of LLINs depends to attrition, physical integrity and insecticidal activity. However, net fabric may play a role in the comfort of the user. Wash resistance represents also an essential factor for LLINs durability. Polyester nets are usually smooth and have a soft touch with a good acceptance. Polyethylene nets have the thickest yarns and highest square meter weights [19]. While, the effective lifespan of polyester nets are longer than polyethylene, these two fabrics do not last for the 3 years recommended by WHO [19]. It is therefore important to assess new net fabric material in terms longer physical durability and efficacy and maybe the opportunity to use this alternative material for binding new insecticides/chemicals.

Bayer CropScience has developed a new deltamethrin-treated LLIN named LifeNet^®^. Technical deltamethrin is incorporated into 100 denier poly-filament polypropylene fibers at the target dose of 8.5 g AI/kg, corresponding to 340 mg of deltamethrin per m^2^ LLIN. This new LLIN is made of soft filament, has a greater mechanical strength, a superior insecticide wash resistance with a short insecticide regeneration time, a better flammability profile and a better environmental profile compared to polyester or polyethylene nets according to the manufacturer [20]. The efficacy of the LifeNet® in experimental huts against free-flying wild mosquitoes was already evaluated under WHOPES supervision. Results of this evaluation showed that LifeNet® fulfilled WHOPES requirements for Long Lasting technology in Phase II [21].

In the present study, LifeNet^®^ was evaluated under WHOPES supervision at community level, in operational conditions, through a prospective longitudinal study using a WHOPES fully recommended LLIN (PermaNet® 2.0) as a positive control. The primary outcomes were physical integrity, insecticidal activities, perception, acceptance, use and survivorship of Lifenet®.

## 2. Material and methods

### 2.1. Study area and villages selection

The study was carried out in the Ouidah Kpomasse Tori health district (hereinafter referred to as the OKT health district), one of 34 health districts in Benin. This district has essentially a subequatorial climate with two dry seasons (August-September and December-March) and two rainy seasons (April-July and October-November). The average annual rainfall is around 1200 mm, of which 700–800 mm come in the first rainy season and 400–500 mm come in the second rainy season. The average monthly temperatures vary between 27 and 31°C. The northern part of the OKT health district is made of a plateau that drops into the Couffo valley and the Allada depression, while the southern part is watered by several ramification arms of Lake Toho. The study zone is totally cleared of its original equatorial forest and is currently characterized by bushes and isolated trees, associated with more or less densely populated areas and oil palm trees [22]. Malaria transmission is described as meso-endemic with an entomological inoculation rate of 5.3 infective bites per person per year. The main malaria vectors are *Anopheles gambiae s*.*l*. and *Anopheles funestus*. Resistance to pyrethroids has shown to be moderate [22].

Villages of the OKT health district were visited to assess their eligibility for the study. The main criteria for eligibility were the size and the accessibility of the villages. The eligible villages were randomly selected in collaboration with the NMCP of Benin. After a pre-test of the census questionnaire, the census was conducted in eligible villages. The census started in the first eligible village randomly selected and continued in the following villages until the 3000^th^ sleeping unit was reached. All the sociodemographic data collected in the selected villages were centralized in the database through a double entry process.

### 2.2. Net characteristics

The two types of nets in the study have the same color (white). Roof and faces of nets were rectangular. Regarding nets size, PermaNet® was 190 cm width, 180 cm length and 200 cm height, while LifeNet® was 190 cm width, 180 cm length and 150 cm height. LifeNet® is a long lasting insecticidal polypropylene nets with a target deltamethrin dose of 340 mg / m^2^ and PermaNet® is a long lasting insecticidal polyester nets with a target deltamethrin dose of 55 mg / m^2^.

### 2.3. Communication

The achievement of this community-based Phase III study required a good understanding of its objectives and content by both the authorities and the communities. In collaboration with the NMCP, we built up a communication plan which was then validated by administrative and health authorities through several workshops. In accordance with the communication plan, an opening ceremony was organized in the presence of all partners working on malaria control in Benin, health and administrative authorities and local community stakeholders. Regular meetings with the local authorities were also organized to keep them informed of the progress of the project and the schedule. Before the distribution of LLINs, informative meetings were held with the stakeholders to share information on the distribution procedure and the standard utilization of bed nets.

### 2.4. Study design

The trial was a prospective longitudinal, cluster-randomized controlled trial with households as the unit of observation. At baseline, all enrolled houses were numbered and socio-demographic and economic data, including age, sex, occupation, education, sleeping habits and number of spaces were collected using a standardized questionnaire. The global positioning system (GPS) coordinates of each house was recorded at the front door using Garmin device. Clusters of households were selected for inclusion so that the whole community is covered with LLINs in the selected villages.

LifeNet® and PermaNet® nets were labeled with a unique number randomly chosen from 1 to 4000 and repacked into unlabeled bags to keep the “double blindness” of the study as far as possible. In each household, the same type of LLINs was distributed. Neither the beneficiary community nor the field workers know what type of nets are being distributed. All householders were provided with LLINs in accordance with national policy of Benin, which is one LLIN for two people. All sleeping units were covered in the selected villages.

The study started in June 2014, during the raining season. At the beginning of the study (0 M) and every 6 months thereafter (6, 12, 18, 24, 30, 36 M), around 50 households where each type of LLIN was distributed were randomly selected. One LLIN of each selected household was withdrawn, replaced with a new net of the same type and sent to the laboratory for physical integrity assessment, bio-assays and chemical assays. All collected LLINs were drawn from the net master list by the principal investigator. Chemical assays were performed on LLINs collected at 0, 12, 24 and 36 M while physical integrity assessment and WHO cone bio-assays were carried out on LLINs collected at 0, 6, 12, 18, 24, 30 and 36 M. Based on cone bio-assays results, all nets that did not meet the efficacy criteria of ≥95% knock-down rate after 60 minutes or a mortality of ≥80% after 24 h of 3 minutes’ exposure were subjected to a tunnel test. Mortality and blood feeding inhibition in tunnel tests were determined.

To assess any adverse effects, 50 LLINs of each type were selected and there users were interviewed 1 week and 1 months post distribution. To assess owners’ perception on LLINs, LLINs acceptance, attrition rate and use rate, 250 LLINs of each type were randomly selected every 6 months (6, 12, 18, 24, 30, 36 M) and heads of households where LLINs were distributed were interviewed. LLINs were not withdrawn from households. The LLINs sampling scheme during the trial is summarized in **Table 1**.

**Table 1:** Summary of LLINs sampling scheme during the study.

Time point	Number of LLINS of each type withdrawn and conveyed to laboratory for chemical assays + physical integrity assessment + bioassays	Number of LLINS of each type withdrawn and conveyed to laboratory for physical integrity assessment + bioassays	Number of LLINS of each type (not withdrawn) and whose owners were interviewed for use and attrition (survivorship) assessment	Number of LLINS of each type (not withdrawn) and whose owners were interviewed for side effects assessment
**0**	50			
**1 week**				50
**1 month**				50
**6 months**		50	250	
**12 months**	50		250	
**18 months**		50	250	
**24 months**	50		250	
**30 months**		50	250	
**36 months**	50		250	

### 2.5. LLIN treatments

#### 2.5.1. Chemical assays

From each LLIN, four samples (30 cm x 30 cm) were cut as indicated in WHOPES guidelines and rolled up and placed in labeled clean aluminum foil [23]. The samples were kept at +4°C temperature prior to their shipment to WHOPES for chemical assays. The samples from each LLIN were combined to provide the average target concentration of the insecticide in each LLIN.

#### 2.5.2. Cone bioassays

For the cone bioassays, five samples (25 cm x 25 cm) were cut from each LLIN. The standard WHOPES procedure was used for evaluation of insecticidal effect of LLINs [23]. Bioassays were performed using 2–5 days old non-blood fed, laboratory maintained susceptible *Anopheles gambiae* Kisumu strain. Ten females were exposed to each netting sample in standard WHO cones fixed with a plastic manifold for 3 minutes and then held for 24 h in paper cups with cotton wool soaked with 10% sugar solution. Knockdown was recorded after 60 minutes and mortality after 24 h. For each netting sample, the test was replicated. The total number of mosquitoes exposed per net was 100. Mosquitoes exposed to untreated nets were used as negative controls in each round of assays. All bioassays were carried out at 27±2°C and 80 ± 10% relative humidity. Based on cone test results, tunnel tests were carried out on LLINs which did not reach the WHO efficacy criteria (mortality rate < 80% or knockdown rate after 60 minutes < 95%).

#### 2.5.3. Tunnel tests

Tunnel tests were carried out in the laboratory as described in the WHOPES guidelines [23] using a guinea pig as bait. The experiment began at 18:00 and end at 09:00 the following morning. Briefly, one hundred 5–8 days old non blood fed Kisumu mosquitoes were introduced in a glass tunnel (square section 25 cm x 25 cm) 60 cm length [23]. During the experiment, the tunnel was maintained at 27°C ± 2°C and 80% ± 10% relative humidity under subdued light. One tunnel with untreated netting was used as a negative control. At 09:00 the following morning, mosquitoes were removed by using a suction glass tube and counted separately from each section of the tunnel. Mortality and blood feeding rates were recorded. Overall mortality was measured by pooling mortality rates from the two sections of the tunnel. Blood feeding inhibition was assessed by comparing the proportion of blood fed females (alive or dead) in treated and control tunnels. As blood feeding rates in controls have a considerable impact on mortality in the presence of treated samples (i.e. the host seeking behavior increases the chance of contact with treated fabric), a minimum cut off of 45% blood feeding rate in controls was established to validate tunnel tests.

#### 2.5.4. LLINs physical integrity assessment

Size and distribution of holes on LLINs were evaluated in the laboratory to assess the physical integrity of LLINs. LLINs were draped over a frame and the number of holes of different sizes according to location on the net (top, upper side, lower side) was counted. For hole index calculation, holes were weight 1; 23; 196 and 576 respectively for holes with 0.5–2.0; 2–10; 10–25 and >25 cm. Based on hole index, LLINs were classified as good (hole index < 64), damage (64 < hole index < 643) or too torn (hole index ≥ 643) [24]. The hole index was determined using the following formula:

Hole index (hi) = (A × no. of size-1 holes) + (B × no. of size-2 holes) + (C × no. of size-3 holes) + (D × no. size-4 holes), with A = 1, B = 23, C = 196, and D = 576, corresponding to the estimated areas, assuming that the size of holes in each category is equal to the midpoints.

### 2.6. Assessment of community acceptance, practices, net-attrition and side effects

#### 2.6.1. Quantitative cross-sectional surveys

Assessment of adverse effects, if any, and perception among LifeNet® and PermaNet® users was carried out using a structured questionnaire. A team of two socio-anthropologists has visited the selected householders one week and one month after LLIN distribution. The Principal Investigator informed the Medical Officer of the area about possible reporting of side effects and to provide medical care, as necessary. The Principal Investigator also collected data of such events from the Medical Officer.

At 6 M, 12 M, 24 M, 30M and 36 M, surveys were conducted by door-to-door visits of 500 randomly selected households with remaining LLINs (250 of each type) to record physical presence/absence, to estimate the annual attrition rate, besides information on people perceptions and practices. During the interview, the adult participants were asked to assess net utilization patterns (including early morning observations), method and number of washes, type of soap used.

#### 2.6.2. Qualitative longitudinal survey

One anthropologist based in the village was in charge of the qualitative survey leading to an anthropological analysis of social representations, everyday life and household nets handling (especially for technical procedures: installing/uninstalling, washing, drying and mending practices, etc.). Perception, understanding, processing and transmission of information and educational messages related to LLINs for both people in villages and professional or administrative staff involved in this evaluation were recorded. This approach allowed to analyze the deformation, reinterpretations and impacts of an initial message, especially through the linguistic and cultural prisms. Such approach includes the analysis of the design process messages and information sessions and health education. The completion of the qualitative survey relies on semi-structured interviews, focus groups and participant observation. Visual anthropology was used, especially for technical acts of daily life related to the nets and health education sessions.

### 2.7. Ethical approval for the study

The protocol of the study was submitted in Benin to the National Ethics Committee for Research and the ethical clearance was received (ethical clearance N° 017 of 28th June 2012) and renewed annually. Participants were adults responsible of each household of villages selected for the study. Written free and informed consent was obtained from each participant after the study had been presented to him. The study has considered the ethical issue of protecting people’s rights, possible inconveniences caused to them and protecting infringement of individual privacy during the study and more specifically during census and sociological surveys.

### 2.8. Statistical analysis

Data analysis was carried out with R software, version 3.6.3. Mosquito mortality rates, Knock-down after 60 minutes (KD60) rates, blood-feeding rates, passage rates, proportion of LLINs in good physical condition, median holes indexes at each time point, proportion of LLINs reaching WHO efficacy criteria and LLIN use rates were analysed using a binomial Generalized Linear Mixed Model (GLMM) with villages and households as random intercepts and the total tested as weights. Rates were estimated with their 95% confidence intervals and compared using khi^2^ test. The LLIN survivorship was estimated using Kaplan-Meier method. Overall and for each LLINs type, the proportion of LLINs in good physical condition were compared using Khi^2^.

## 3. Results

### 3.1. Household characteristics and net distribution/coverage

Three thousand sleeping units belonging to three villages (Cada II, Satré, Zounmé) were selected for the trial. At the beginning of the trial, the population size in the selected villages was 4716 with 1603 households and 3204 sleeping units (Table 2). The population age pyramid is shown on supplementary file. Overall, 3095 LLINs (1600 LifeNet® and 1495 PermaNet®) were distributed to 1549 households in the three villages. Household and sleeping unit net coverage rates were both equal to 96.6%. Almost all sleeping units without LLINs were either damaged or unoccupied because their inhabitants moved away. Tree householders did not want to take the LLINs.

**Table 2:** Population size and LLIN distribution and coverage in each village.

Villages	Cada II	Satre	Zounmé	Total
**Population size**	1639	1681	1396	4716
**Households**	575	563	465	1603
**Household LLIN coverage (%)**	550 (95.6)	542 (96.3)	457 (98.3)	1549 (96.6)
**Sleeping units**	1084	1162	958	3204
**Sleeping unit LLIN coverage (%)**	1049 (96.8)	1136 (97.8)	910 (95.0)	3095 (96.6)

### 3.2. Insecticidal activities of LLINs

#### 3.2.1. Chemical content

**Fig 1:**
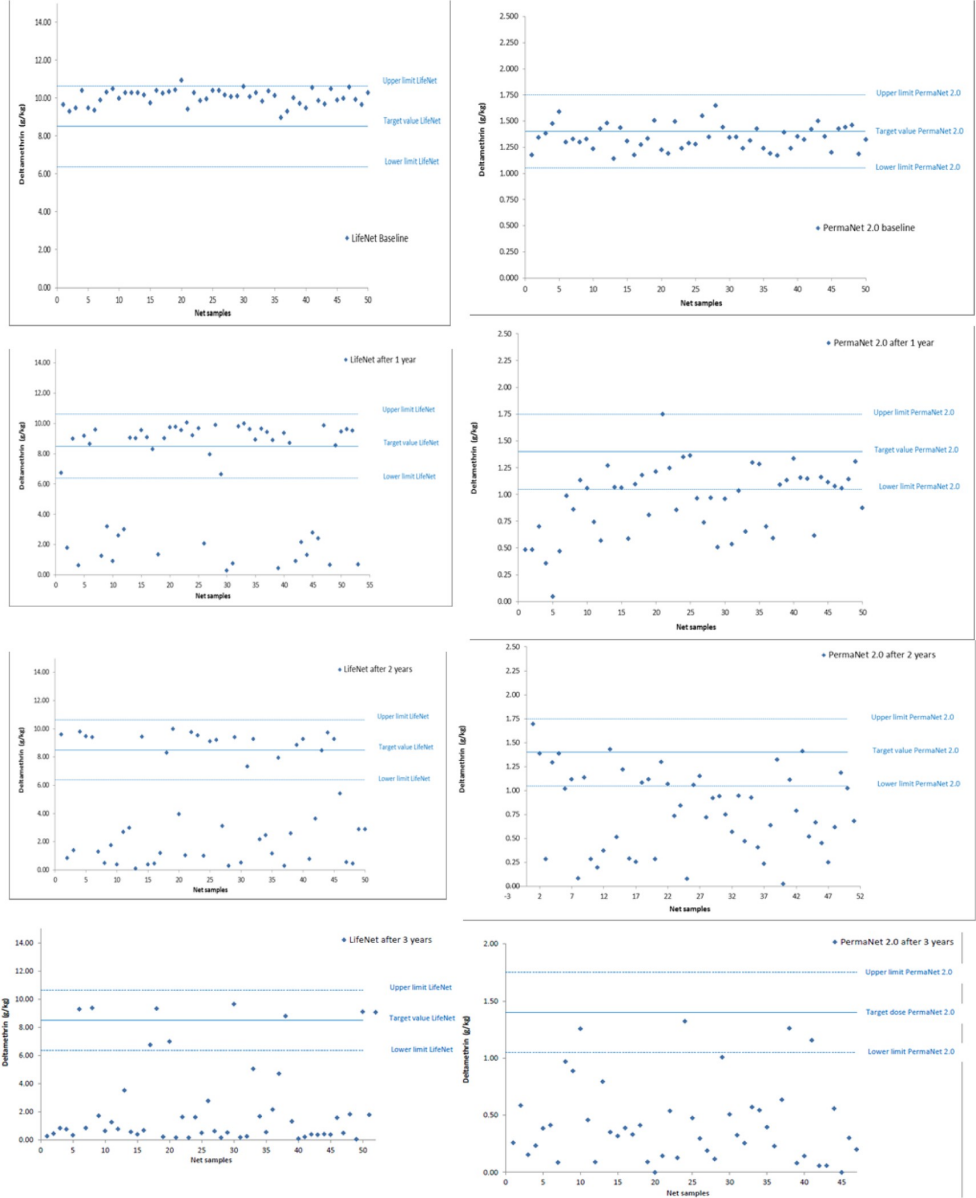
Insecticide content (g/kg) in LifeNet and PermaNet 2.0 at 0, 12, 24 and 36 months after distribution.

Results from chemical analysis are shown on Fig 1. At the beginning of the trial (0 M), all unused LifeNet^®^ LLINs sampled (50/50, 100%) had an average active deltamethrin content of 10.03 ± 0.12 g/kg corresponding to 439.8 ± 6.7 mg/m^2^, which is above the target dose of 374 mg/m^2^ but within the limit allowed. Only one LifeNet® LLIN had an active deltamethrin content above the maximum limit allowed. Likewise, all (50/50, 100%) unused PermaNet^®^ LLINs were within their target chemical dose of 1.34 ± 0.03 g/kg corresponding to 59.7 ± 1.5 mg/m^2^. After 12, 24 and 36 M of use under daily household conditions, 64.2% (34/53), 40% (20/50) and 17.3% (9/52), respectively of LifeNet^®^ LLINs sampled were still within the limits of their initial target value. With PermaNet^®^ LLINs 50% (25/50), 35.3% (18/51) and 8.5% (4/47) of nets sampled, respectively after 12, 24 and 36 M, were still within the limits of the initial target dose.

#### 3.2.2. Bio-efficacy

**Fig 2:**
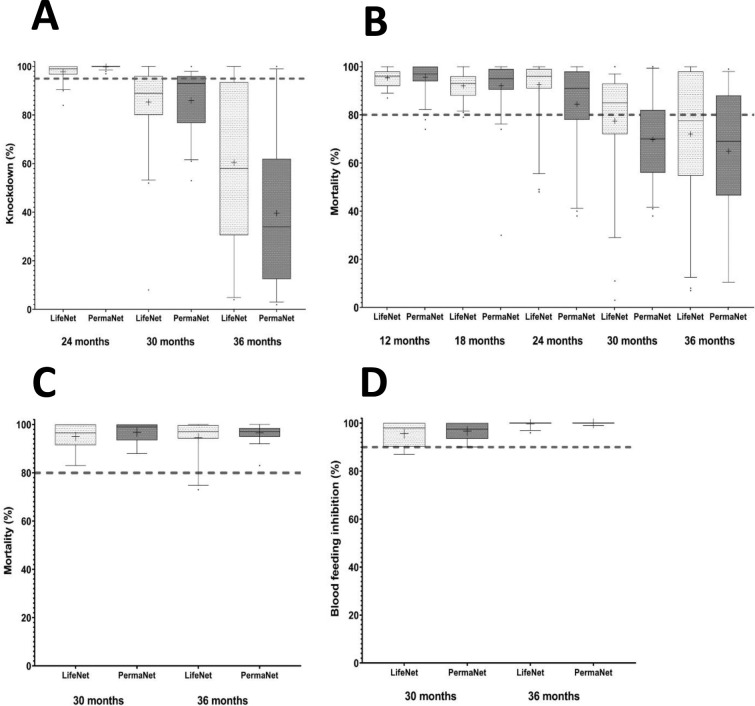
Bio-efficacy of LifeNet and PermaNet LLINs against *An*. *gambiae* Kisumu strain. (A) and (B) show knockdown and mortality rates using cone bio-assays, while (C) and (D) display mortality and blood feeding rates using tunnel tests. Boxes display the median value, 25th and 75th percentiles. The whiskers show the 5th/95th percentiles and the dots indicate the outliers. Dashed lines indicate threshold of WHOPES efficacy criteria.

From 6 M to 36 M, 615 LLINs were sampled and tested (Table 3). Results from WHO cone bio-assays are presented on Fig 2. The proportions of LLINs that fulfil the WHOPES criteria (≥95% knockdown or ≥80% mortality) decreased over time. After 30 M, 73% of LifeNet^®^ LLINs complied with these criteria compared to 66% of PermaNet® LLINs, but this difference was not significant (p = 0.62). After 36 M, 42% of LifeNet^®^ LLINs still complied with the WHO criteria, while only 27% of PermaNet® nets did with no significant difference (p = 0.25).

**Table 3:** Proportion of LLINs meeting WHOPES efficacy criteria (knockdown ≥ 95% or mortality ≥ 80%) according to cone tests.

	LifeNet			PermaNet			P value
	N LLINs tested	LLINs Proportion (%)	95% CI	N LLINs tested	LLINs Proportion (%)	95% CI	
**6 months**	52	100	-	54	100	-	1
**12 months**	53	100	-	51	100	-	1
**18 months**	50	100	-	50	97.9	[87.5–99.9]	0.99
**24 months**	50	96	[85.1–99.3]	51	100	-	0.47
**30 months**	51	72.6	[58.0–83.7]	50	66	[51.1–78.4]	0.62
**36 months**	54	41.7	[27.9–56.7]	49	28.6	[17.0–43.5]	0.25

All LifeNet® and PermaNet® LLINs that didn’t reach WHOPES efficacy criteria after WHO cone bio-assays were subject to/underwent tunnel tests. Mortality and blood-feeding inhibition rates under tunnel tests were around 100% for both LLINs at 30 and 36 M (Fig 2), suggesting that all these nets actually met WHOPES efficacy criteria.

#### 3.2.3. Physical integrity/condition of LLINs

**Fig 3:**
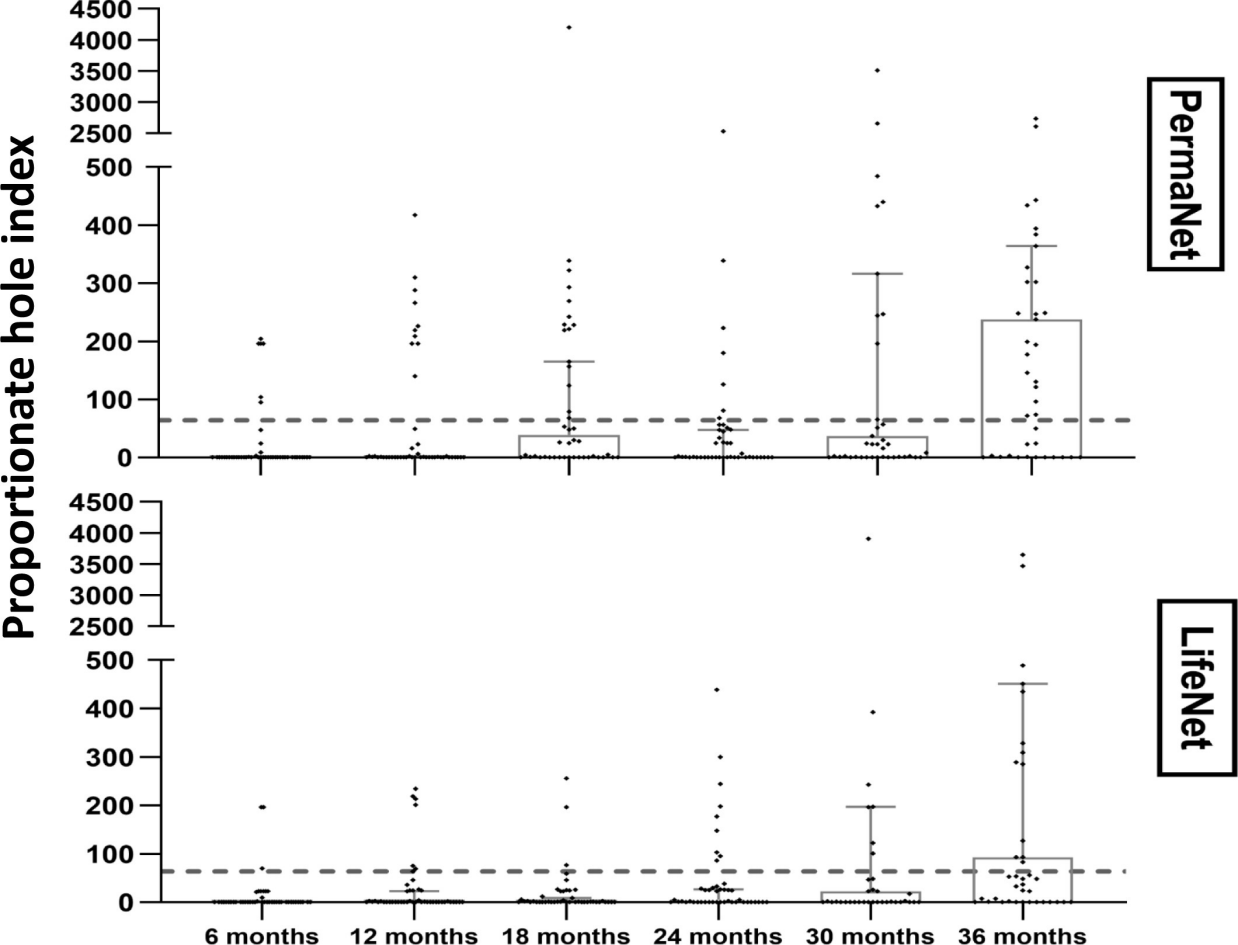
Physical condition of LifeNet and PermaNet LLINs based on the proportionate hole index (pHI). Box plot indicating median and 95% confidence interval, dashed line = threshold of good net condition (pHI ≥64).

Proportional hole indexes of both LLINs during the trial are shown on the Fig 3. The proportion of LLINs in good physical condition (hole index < 64) at each time is presented in the Table 4. The proportion of LLINs in good physical condition decreased significantly at 36 months compared to previous time points (p = 0.03 for LifeNet® and p = 0.01 for PermaNet®). At each time, no significant difference was observed between the proportions of two LLIN types in good physical condition (p>0.20). Similarly, overall, no significant difference was observed when comparing the good physical integrity of PermaNet® to LifeNet® as a function of time (Khi² test P = 0. 42). However, between time points, a significant difference was observed for each LLIN type (Khi^2^ test P<0. 001).

**Table 4:** Proportion of LLINs in good physical condition over time.

	LifeNet			PermaNet			P value
	N LLINs tested	LLINs Proportion (%)		N LLINs tested	LLINs Proportion (%)		
**6 months**	52	92.3	[91.3–93.3]	54	81.5	[80.0–82.9]	0.66
**12 months**	53	77.4	[75.8–78.9]	51	74.5	[72.8–76.2]	0.90
**18 months**	50	84.0	[82.6–85.4]	50	56.0	[54.1–58.0]	0.20
**24 months**	50	74.0	[72.3–75.7]	51	73.1	[71.4–74.8]	0.97
**30 months**	51	57.1	[55.2–59.1]	50	51.0	[49.1–52.9]	0.74
**36 months**	54	46.2	[44.3–48.0]	49	30.6	[28.8–32.5]	0.28

### 3.3. LLINs use and survivorship

In the first 6 M of the trial, 76% of LifeNet^®^ and 81.8% of PermaNet^®^ owners declared to use their nets (Table 5). This dropped to 20% for LifeNet^®^ and 45% for PermaNet^®^ after 18 M, before rising again to 72.7% and 90.9% after 36 M for LifeNet® and for PermaNet®, respectively.

**Table 5:** Proportions of LLIN usage throughout the study period.

	LifeNet				PermaNet					
	Responders	N LLIN users	% LLIN use rate	95% CI	Responders	N LLIN users	% LLIN use rate	95% CI	P value
**6 months**	121	92	76.0	[63.4–83.6]	143	117	81.8	[75.5–88.1]	0.69
**12 months**	98	62	63.3	[53.7–72.8]	109	80	73.4	[65.1–81.7]	0.50
**18 months**	45	9	20.0	[08.3–32.0]	40	18	45.0	[29.6–60.4]	0.08
**24 months**	163	86	52.8	[45.1–60.4]	140	80	57.1	[49.0–65.3]	0.68
**30 months**	24	20	83.3	[68.4–98.2]	31	25	80.7	[66.7–94.6]	0.93
**36 months**	66	48	72.7	[62.0–83.4]	55	50	90.9	[83.3–98.5]	0.41

**Fig 4:**
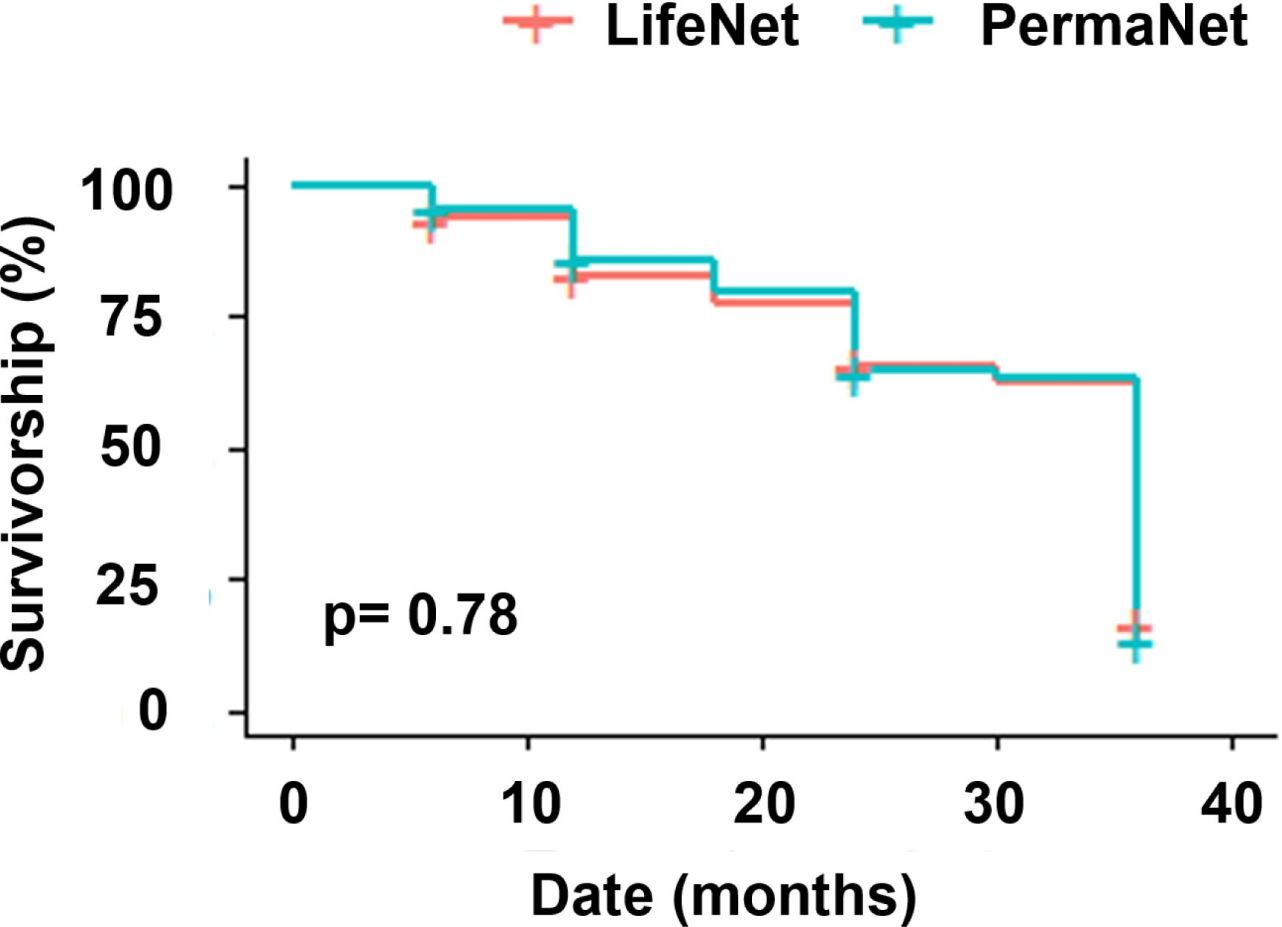
Survivorship of LifeNet and PermaNet LLINs.

The survivorship stayed above 75% for both LLINs in the first 18 M but dropped to around 25% for both LifeNet^®^ (21%) and PermaNet^®^ (26%) after 36 M (Fig 4).

### 3.4. Side effects

Adverse events declared by net users one week (102) and one month (100) after LLIN distribution are summarized in Table 6. The proportion of LifeNet® users who declared at least 1 side effect (86.5%) after one week of use was significantly higher than that of PermaNet® users (58%) (Khi^2^ test, p <0.05). One month after, this proportion has significantly reduced for both LLINs and there was no significant difference between LifeNet® (32%) and PermaNet® (22%) LLIN users (khi^2^ test, p>0.05). The most frequently declared adverse events were burning and itching skin (Table 7). LifeNet® users were those who complained the most of burning (81.5%) and itching (45.7%) one week after LLIN distribution compared to PermaNet® users (51.4 and 20.3%, respectively) (OR > 3, p < 0.05). This trend remained one month after LLINs distribution, even if it was significantly reduced (khi^2^test, p < 0.05) and several adverse events disappeared. Eye irritation only persisted among LifeNet® LLIN users (7.9%) one month after LLIN distribution.

**Table 6:** Proportion of LLIN users who declared at least one side effect at 1 week and 1 month after LLIN distribution.

	LifeNet				PermaNet				P value
	N	Complainers	%	95% CI	N	Complainers	%	95% CI	
**1 week**	52	45	86.5	[77.26–95.82]	50	29	58.0	[44.32–71.68]	0.20
**1 month**	50	16	32.0	[19.07–44.93]	50	11	22.0	[10.52–33.48]	0.40

**Table 7:** Side effects declared by participants at 1 week and 1 month after LLIN distribution.

Side effects	LifeNet			PermaNet				
1 week	N	N complainers	%	N	N complainers	%	OR	P value
Itching	81	37	45.7	74	15	20.3	3.28 [1.53–7.29]	0.001
Burning	81	66	81.5	74	38	51.4	4.12 [1.91–9.24]	<0.001
Sneezing	81	5	6.2	74	3	4.1	1.55 [0.29–10.36]	0.722
Liquid discharge from	81	9	11.1	74	2	2.7	4.62 [0.88–43.86]	0.059
Headache	58	4	6.9	55	1	1.8	3.95 [0.38–20.38]	0.365
Nausea	81	0	0.0	74	0	0.0	NA	NA
Eye irritation	81	13	16.0	74	5	6.8	2.62 [0.82–9.91]	0.083
Tearing	81	0	0.0	74	0	0.0	NA	NA
Bad smell	81	10	12.3	74	1	1.4	10.30 [1.40–45.73]	0.010
**1 month**								
Itching	76	11	14.5	74	4	5.4	2.94 [0.82–2.30]	0.1004
Burning	76	19	25.0	74	14	18.9	1.42 [0.61–3.39]	<0.0001
Sneezing	76	0	0	74	0	0	0	NA
Liquid discharge from	76	0	0	74	0	0	0	NA
Headache	76	0	0	74	0	0	0	NA
Nausea	76	0	0	74	0	0	0	NA
Eye irritation	76	6	7.9	74	0	0	10.58 [1.19–25.48]	0.0283
Tearing	76	0	0	74	0	0	0	NA
Bad smell	76	0	0	74	0	0	0	NA

### 3.5. Community acceptance and practices

Anthropologists based in the three selected villages for the study were in charge of the qualitative study leading to an anthropological analysis of social representations, perception, understanding, processing and transmission of information and educational messages related to the LLINs, for both people in villages and professional or administrative staff involved in this evaluation.

#### 3.5.1. Communication, sensibilization and population adherence to the project

Before LLINs distribution, public awareness campaigns were carried out in the 3 villages. During these campaigns, the objectives, the modalities of the study and LLINs distribution procedures were exposed to populations in presence of their chiefs. It was clearly mentioned that the success of the trial relies on the withdrawal of LLINs in use in their households to be replaced by LLINs to be studied. Populations participated massively to the LLINs distribution indicating that having a free brand new LLINs was of great importance.

#### 3.5.2. LLINs perception

Along LLINs distribution campaign, we observed that the majority of the people checked the fiber features of the LLINs. Fiber features of both Lifenet® and Permanet® were appreciated by populations. Populations considered that these LLINs had a soft texture compared to other LNs they received from the National Malaria Control Program (NMCP). In contrast, populations complained that both Lifenet® and Permanet® did not have any device (string and nail) to facilitate their installation.

Users also noted that Lifenet® was less high than Permanet® and other LLINs received from the NMCP. This low height of Lifenet® led to installation difficulties. Because of the small size of Lifenet®, some of users declared a confinement feeling.

#### 3.5.3. Side effects

One week after LLINs distribution, some participants declared that they uninstalled their Lifenet® LLINs previously installed due to perceived side effects. In response to side effects, some of Lifenet® LLIN users decided to hang their LLINs outside for several days waiting for the insecticide to evaporate and side effects to disappear. In the same context, other Lifenet® users also declared that they washed their LLINs several times. After such spontaneous procedures, these Lifenet® users however reinstalled their LLINs in their house.

#### 3.5.4. Utilization procedures

During the qualitative longitudinal surveys, we also noted interesting information about the LLINs installation and utilization processes. In large households, both Lifenet® and Permanet® LLINs were installed permanently. In contrast, in small households, LLINs were installed at dusk and removed in the morning. In this last case, LLINs removed were sometimes stored during the daytime in inappropriate sites like on drying string (i.e directly exposed to sun light for several hours), or on some sharp objects which might damage LLINs.

#### 3.5.5. Washing procedures

In the study area, the floor of houses was local red clay. Consequently, LLINs were rapidly dirty and therefore regularly washed. After washing, we observed numerous LLINs were hung under sun light for many hours despite populations were advised not to do so during the public awareness campaigns before LLINs distribution.

#### 3.5.6. Population displacements

Attrition relied mainly on numerous people displacement. Hereafter are described some of declared reasons explaining them. 1) In dry season for example, young farmers from the villages of the study area moved to Nigeria (neighboring country) for economic reasons. 2) Some of LLINs users are pupils. During the trial, some of them obtained the high school diploma and then went to Cotonou, the economic capital of Benin, to go to high School. 3) Sometimes, people declared that they gave their LLINs to somebody living outside the study area. For example, some mothers sent their LLINs to their daughters who gave birth.

## 4. Discussion

The performance of the interim WHOPES recommended LLIN LifeNet® was evaluated in comparison with the fully WHOPES recommended LLIN, PermaNet®, as positive control. The evaluation was carried out under field conditions during a three-year trial. The performance of LifeNet® LLINs was equal to and sometimes greater than that of PermaNet® nets.

LLINs are useful and effective tools for preventing malaria that combine both chemical and physical barriers [23]. The requirements for a candidate LLIN to obtain fully WHOPES recommendation is that, at the end of three years use on field, at least 80% of the sampled LLINs retain bio-efficacy (i.e. ≥95% mosquito knockdown rate or ≥80% mortality) with a standard WHO cone bio-assay or with a tunnel test (≥80% mortality or ≥90% blood feeding inhibition) [7]. Based on those criteria, LifeNet® LLINs meet WHO efficacy criteria in the present phase III study with 100% of effective nets after three years under daily use. LifeNet® nets could then be added to the arsenal of LLINs distributed by the NMCP of Benin for malaria prevention.

Despite a higher deltamethrin concentration on LifeNet® nets (up to 7 folds higher) compared to PermaNet® nets, no significant difference in bio-efficacy was observed between the two types of LLIN during the 3-year study period. Such result suggests that a greater concentration of insecticide on a given net does not systematically lead to a significant increase in its residual activity. On the contrary, the increase of insecticide on LLINs seems to amplify side effects as noticed for LifeNet® nets in the present study. The real interest of increasing the concentration of deltamethrin on LifeNet® mosquito nets become quite questionable, since side effects can lead to certain bad practices as demonstrated in this trial. For instance, some participants, mostly LifeNet® users, uninstalled their nets a few days after their installation, washed them several times and hung them outside under the sun for many days before reinstalling them, owing to the side effects. Manufacturers of LLINs should therefore work to reduce side effects as much as possible to avoid non-adherence or bad practices by the target population.

Regarding physical integrity, more than the three quarters of the nets were still in good physical condition 6 months after distribution, hence theoretically more effective in protecting homeowners from mosquito bites. However, the proportion of LLINs in good physical condition decreased significantly at 36 months (P < 0.05). This decrease would probably be due to the maintenance, washing or lifespan of the LLINs. This suggests that whatever the fabrics (polyester, polyethylene or polypropylene), their lifespan does not exceed the 3 years recommended by the WHO [4].

PermaNet® was more used than LifeNet® in the study area. This could be explained by the higher insecticidal effect of LifeNet®. LifeNet® users were reluctant to use this type of net, which was allocated to them, probably because of the reported side effects. In addition, the overall survivorship of LLINs was estimated at 21% for LifeNet® and 26% for PermaNet® at 36 months. However, between 30 and 36 months, the significant drop in survival observed could be due to the high proportion of LLINs in poor physical condition at 36 months.

In the present study, most of people checked the netting fibers and did appreciate both LifeNet® and PermaNet® nets for their softness compared to those distributed by the NMCP, indicating that net fabric counts for LLINs acceptability. However, WHOPES criteria for recommending LLINs seem to only take into account the insecticidal efficacy after three years of use.

The durability of LifeNet® nets was comparable to that of PermaNet® after 36 M, despite the manufacturer claims that polypropylene-made LLINs (LifeNet®) will have a greater durability for household use over polyester-made LLINs such as PermaNet® nets [20]. The physical durability of the fabric being the main factor determining the lifespan of LLINs, manufacturer of LifeNet® needs to work for improving durability of this LLINs to make it greater than that of polyester nets as it is stated.

Some limitations of the present study merit to be mentioned. The bio-efficacy tests were done with the susceptible mosquitoes from the laboratory. These tests should also have been done with a resistant population to assess resistance selection pressure of LLINs, seen the high insecticide concentration in LifeNet® compared to PermaNet®. The variation in responders to assess net usage is a big issue. In addition to that, the low number of responders probably influenced the results of this study. It would be better to survey users of 250 LLINs in order to increase the number of responders at each time point.

## 5. Conclusion

The bio-efficacy and lifespan of LifeNet® nets were expected to be greater than those of PermaNet® after three years, owing to their high concentration of deltamethrin and the fabric (polypropylene). In this study, there were no significant difference in terms of bio-efficacy and durability between LifeNet® and PermaNet® nets. Both LLINs were well accepted by the population with use rates over 70% after 3 years of survey. However, side effects were more frequent with LifeNet® than PermaNet® 2.0.

## Supporting information

S1 File (ZIP)
